# Estrogen-Induced Hypermethylation Silencing of *RPS2* and *TMEM177* Inhibits Energy Metabolism and Reduces the Survival of CRC Cells

**DOI:** 10.3390/cells15020124

**Published:** 2026-01-09

**Authors:** Batoul Abi Zamer, Bilal Rah, Wafaa Abumustafa, Zheng-Guo Cui, Mawieh Hamad, Jibran Sualeh Muhammad

**Affiliations:** 1 Department of Basic Medical Sciences, College of Medicine, University of Sharjah, Sharjah 27272, United Arab Emirates; 2 Research Institute of Medical and Health Sciences, University of Sharjah, Sharjah 27272, United Arab Emirates; 3 Department of Environmental Health, University of Fukui School of Medical Sciences, Fukui 910-1193, Japan; sai@u-fukui.ac.jp; 4 Department of Medical Laboratory Sciences, College of Health Sciences, University of Sharjah, Sharjah 27272, United Arab Emirates; 5 Department of Biomedical Sciences, College of Medicine and Health, University of Birmingham, Birmingham B15 2TT, UK

**Keywords:** estrogen, colorectal cancer, estrogen receptors, mitochondria, metabolism, oxidative phosphorylation

## Abstract

**Highlights:**

**What are the main findings?**

- Estrogen (E2) acts through ERα to suppress colorectal cancer cell growth by disrupting mitochondrial function and the oxidative phosphorylation (OXPHOS) pathway.
- The OXPHOS-associated genes RPS2 and TMEM177 are overexpressed and hypomethylated in CRC tissues, negatively correlate with ERα expression, and are epigenetically silenced by E2.

**What are the implications of the main findings?**

- Silencing of RPS2 or TMEM177 phenocopies E2 treatment, leading to mitochondrial membrane depolarization, impaired OXPHOS, and reduced CRC cell viability.
- These findings reveal an ERα-dependent epigenetic–metabolic vulnerability in colorectal cancer that may be exploited for hormone- or epigenetic-based therapeutic strategies.

**Abstract:**

Estrogen (E2, 17β estradiol) is recognized for its regulatory role in numerous genes associated with energy metabolism and for its ability to disrupt mitochondrial function in various cancer types. However, the influence of E2 on the metabolism of colorectal cancer (CRC) cells remains largely unexplored. In this study, we examined how E2 affects mitochondrial function and energy production in CRC cells, utilizing two distinct CRC cell lines, HCT-116 and SW480. Cell viability, mitochondrial function, and the expression of several genes involved in oxidative phosphorylation (OXPHOS) were assessed in estrogen receptor α (ERα)-expressing and ERα-silenced cells treated with increasing concentrations of E2 for 48 h. Our results indicated that the cytotoxicity of E2 against CRC cells is mediated by the E2/ERα complex, which induces disturbances in mitochondrial function and the OXPHOS pathway. Furthermore, we identified two novel targets, *RPS2* and *TMEM177*, which displayed overexpression, hypomethylation, and a negative association with ERα expression in CRC tissue. E2 treatment in CRC cells reduced the expression of both targets through promoter hypermethylation. Treatment with 5-Aza-2-deoxycytidine increased the expression of *RPS2* and *TMEM177*. This epigenetic effect disrupts the mitochondrial membrane potential (MMP), resulting in decreased activity of the OXPHOS pathway and inhibition of CRC cell growth. Knockdown of *RPS2* or *TMEM177* in CRC cells resulted in anti-cancer effects and disruption of MMP and OXPHOS. These findings suggest that E2 exerts ERα-dependent epigenetic reprogramming that leads to significant mitochondria-related anti-growth effects in CRC.

## 1. Introduction

Colorectal cancer (CRC) ranks as the third most prevalent cancer worldwide, with over 1.84 million new cases reported annually [1,2]. Various risk factors are associated with CRC, with age being the most significant [3]. Notably, gender disparities persist across all age groups, with men exhibiting a 30% higher incidence and a 40% higher mortality rate compared with women [4]. Numerous clinical and experimental studies have examined gender as a potential risk factor in CRC. In 1991, the Women’s Health Initiative highlighted the importance of sex hormones, particularly estrogen (E2, 17β estradiol), in reducing CRC progression [5]. However, the precise bias for gender disparity in CRC remains ambiguous.

E2 is the most potent female sex hormone, and its corresponding receptors (ERs) play important roles in oncogenesis, cancer progression and therapeutic response. E2 signaling has been shown to exert diverse effects in cancers of the breast, ovary, uterus, prostate, lung, and liver [6,7,8,9]. In CRC, a progressive reduction in E2 signaling within tumor tissue has been observed as the cancer advances [4]. This decline in E2 signaling may have significant implications for tumor behavior. Recent studies have demonstrated that E2 treatment induces cell cycle arrest and apoptosis in CRC cells [10]. Furthermore, postmenopausal women using oral hormone replacement therapy were reported to have a 63% relative reduction in CRC risk [11]. Suppression of ER expression has also been observed in CRC patients compared with normal individuals, and this suppression correlates with tumor stage, grade, and unfavorable overall survival [12]. However, the precise mechanism underlying the protective effects of E2 in CRC carcinogenesis remains incompletely understood.

Cancer cells undergo altered metabolic reprogramming, a phenomenon commonly referred to as the “Warburg effect”, which has been recognized as a hallmark of many cancers including CRC [13]. The Warburg effect describes the preferential reliance of many cancer cells on glycolysis for ATP production even in the presence of oxygen [14]. However, the relationship between E2 signaling and ATP production remains poorly understood. Previous studies have shown that E2 reprograms breast cancer cell metabolism by increasing glucose uptake and lactate production, thereby enhancing tumor growth and metastasis [15]. In addition, E2 has been demonstrated to alter oxidative phosphorylation (OXPHOS), the primary pathway for ATP generation, and mitochondrial function in cervical cancer cells [16].

Beyond its well-established function, E2 has been shown to exert significant epigenetic effects that modulate the expression of genes involved in metabolism, growth, and cell survival [17]. In breast cancer cells, E2 regulates DNA methyltransferase activity, inhibiting methylation at specific cytosine residues and thereby promoting cancer cell growth and survival [8]. In contrast, in liver cancer cells, E2 exerts protective effects by suppressing iron metabolism through DNA methylation, ultimately leading to enhanced apoptosis [9]. Whether E2 alters energy metabolism in CRC through epigenetic reprogramming, however, remains largely unexplored.

In this study, we sought to elucidate the epigenetic role of E2 in regulating CRC cell survival, mitochondrial activity, and energy metabolism. Our findings demonstrate that E2 inhibits mitochondrial function through epigenetic alteration, thereby reducing the survival of CRC cells.

## 2. Methods

### 2.1. Cell Lines and Treatment

Human CRC cell lines HCT-116 (RRID: CVCL_0291) (stage D, p53 wild-type), and SW480 (RRID: CVCL_0546) (stage B, p53 mutant), were purchased from the American Type Culture Collection (ATCC, Manassas, VA, USA) [18,19]. Both cell lines were mycoplasma-free and authenticated using short tandem repeat (STR) genotyping and verified to be identical with the STR profile in comparing databases. HCT116 and SW480 were selected because they represent late- and early-stage CRC, respectively, and differ in key oncogenic features (e.g., KRAS and TP53 status), allowing evaluation of estrogen signaling across distinct molecular contexts. Both cell lines express detectable ERα, with ERβ expression remaining low and minimally responsive to E2, consistent with previous CRC studies. This renders them suitable models for assessing ERα-dependent responses. Although normal colonic epithelial cell lines such as CCD841CoN provide baseline physiological comparison, our study specifically focused on cancer-selective metabolic vulnerabilities; nevertheless, published data already show that normal colonic epithelium expresses significantly higher baseline ERα and exhibits distinct metabolic programming compared with CRC cells.

Both cell lines were cultured using a complete RPMI-1640 medium (Sigma-Aldrich, St. Louis, MO, USA), supplemented with 2 mM l-glutamine, 1% non-essential amino acids, 100 U/mL penicillin, 100 µg/mL streptomycin, and 10% fetal bovine serum. These cell lines were consistently employed throughout the study. For each experimental run, cells from each cell line were seeded and incubated at 37 °C in a 5% CO_2_ environment for 24 h. Following this, 1 μM propyl pyrazole triol (PPT), a synthetic nonsteroidal agonist of E2 receptor α (ERα) that demonstrates a 400-fold selectivity in comparison to E2 receptor β (ERβ) [20] (Tocris Bioscience, Bristol, UK), or 17-β-estradiol (E2) (Sigma-Aldrich, St. Louis, MO, USA), was introduced at concentrations of 5, 10, or 20 nM, diluted in 70% ethanol, for the specified time frame according to the particular experiment. We note that these concentrations may exceed typical circulating postmenopausal plasma levels; however, in vitro dosing is commonly used to model receptor engagement and downstream signaling, and local tissue exposure may differ from circulating levels. The E2 concentrations used (5–20 nM) fall within ranges commonly employed in mechanistic studies and approximate local microenvironmental estrogen levels reported in several tumor types, including CRC, where intratumoral concentrations may exceed circulating postmenopausal levels [8,9]. Cells designated as control received the same volume of 70% ethanol, serving as the vehicle.

### 2.2. RNA Interference and Gene Knockdown

To achieve knockdown of each target gene, specific small interfering RNAs (siRNAs) (estrogen receptor 1 (*ESR1*) ‘s4824’, Ribosomal protein 2 (*RPS2*) ‘s12254’, Transmembrane Protein 177 (*TMEM177*) ‘s37384’) were procured from Thermo Fisher Scientific (Waltham, MA, USA). These siRNAs were pre-designed and pre-validated to ensure their potency, causing a substantial reduction (90%) in off-target effects. The transfection process utilized Lipofectamine RNAiMAX Transfection Reagent and Opti-MEM Reduced Serum Medium from Life Technologies (Carlsbad, CA, USA). Alongside the knockdown experiments, a negative control, known as siControl, was incorporated. This control comprised stealth RNAi (Life Technologies). Following a 48 h incubation period post-transfection, the cells underwent the required experiments.

### 2.3. Cell Viability Analysis by MTT Assay

CRC cells were seeded into a 96-well cell culture plate (Thermo Fisher Scientific, Waltham, MA, USA), adjusting the density to the desired range (e.g., 5000–10,000 cells per well), and introducing an appropriate volume of cell culture medium. The plate was subsequently placed in a 5% CO_2_ incubator (Thermo Fisher Scientific (Waltham, MA, USA) at 37 °C for overnight incubation, facilitating the cells’ adherence to the surface. To evaluate the viability of CRC cells following different treatments, the (3-4,5-dimethyl-2-thiazolyl)-2,5-diphenyl-2-H-tetrazolium bromide (MTT) assay was employed. Upon the conclusion of the treatment period, a solution of MTT (20 µL) was introduced to each well of the 96-well plates (Thermo Fisher Scientific), and the plates were incubated once again in the incubator for an appropriate duration (e.g., 2–4 h) to allow for the formation of formazan crystals. After this incubation period, the medium containing the MTT solution was carefully aspirated to avoid disturbing the formazan crystals. To solubilize the formazan crystals, 100 µL of dimethyl sulfoxide (DMSO) was added to each well, ensuring proper suspension. The absorbance of the resulting solution was measured at 540 nm using a microplate reader (Agilent Technologies Inc., Santa Clara, CA, USA).

### 2.4. Crystal Violet (CV) Staining

CRC cells were seeded to achieve 40% confluency and were subjected to the designated concentration of E2 or PPT treatments for a specified duration. Following the treatment period, the cells were gently rinsed with warm phosphate-buffered saline (PBS) and then fixed using a solution of citric acid/methanol. Subsequently, staining was performed using a 0.5% solution of CV in formalin at room temperature, allowing the stain to interact with the cells for 20–30 min. Following the staining phase, the cells were washed with tap water and left to air-dry. For quantitative analysis, the stained cells were dissolved in a 1% sodium dodecyl sulfate (SDS) solution at room temperature, ensuring complete dissolution with proper agitation. The absorbance of the solution was measured at 570 nm using a microplate reader (Agilent Technologies Inc.)

### 2.5. Cell Viability Analysis by xCELLigence RTCA System

The xCELLigence real-time cell analysis (RTCA) system was employed for continuous, real-time monitoring of the biological state of adherent cells by assessing their electrical impedance, which is reflected as a cell index (CI) value (Agilent Technologies Inc., Santa Clara, CA, USA). To initiate the experiment, a total of 6 × 10^3^ CRC cells were seeded into each well of a 16-well plate (Thermo Fisher Scientific) known as an E-plate. After a 24 h incubation period post-seeding, the cells were subjected to the appropriate treatment, maintaining it for 120 h. The RTCA process was carried out by measuring the electrical impedance values that the cells induced. These values were automatically recorded at 15 min intervals over 120 h.

### 2.6. Annexin V and 7-AAD Staining

The assessment of apoptosis was conducted through Flow Cytometry, which enabled the detection of phosphatidylserine externalization on the cell membrane surface. After treatment, the cells were harvested using trypsin, and to ensure cell viability and quality, they underwent three washes with cold PBS to eliminate any residual trypsin and cellular debris. A new Falcon tube was used to hold 200 μL of the cell suspension, to which Annexin V-FITC (5 μL) and 7-amino actinomycin D (7-AAD) (5 μL) were added. The mixture was gently mixed and incubated in a dark environment at room temperature for 15 min. Subsequently, Flow Cytometry (BD FACS Aria^TM^ III, Agilent Technologies Inc.) was employed for apoptosis analysis, following the manufacturer’s recommended guidelines. To ensure accuracy, unstained cells as well as single-stained cells with Annexin V-FITC or 7-AAD controls were used.

### 2.7. Glucose Uptake

To assess glucose uptake in CRC cells, a fluorescent glucose analog, namely 2-[N-(7-nitrobenz-2-oxa-1,3-diazol-4-yl) amino]-2-deoxy-glucose (2-NBDG) from Invitrogen (Catalog #N13195), was employed. The procedure followed the manufacturer’s provided instructions. In summary, cells underwent two washes with cold PBS, after which each well received 0.5 mL of glucose-free RPMI mixed with 100 µM of 2-NBDG, prepared in 250 µL of absolute ethanol. Subsequently, the cells were incubated for 45 min at 37 °C. Post-incubation, the cells were collected, washed twice with cold PBS, and then subjected to centrifugation at 1500 rpm for 5 min at 4 °C. The supernatant was removed, and the cell pellet was suspended in 200 µL of ice-cold PBS within individual tubes. To quantify and analyze the glucose uptake, the cells were promptly assessed utilizing a Flow Cytometer (BD FACS Aria^TM^ III, Agilent Technologies Inc.). The analysis was carried out at an excitation wavelength of 465 nm and an emission wavelength of 540 nm.

### 2.8. ROS Production

To quantify the overall cellular levels of Reactive Oxygen Species (ROS), the 2′,7′-Dichlorofluorescin diacetate (DCFDA) dye was employed (Sigma-Aldrich, St. Louis, MO, USA). Following the treatment, the cells were subjected to staining with 15 μM DCFDA prepared in phenol-free RPMI, carried out for 15 min at a temperature of 37 °C within a 5% CO_2_ incubator. As a positive control, one well was treated with 200 μM of H_2_O_2_. The quantification of fluorescence was executed utilizing the Synergy™ HTX Multi-Mode Microplate Reader (Agilent Technologies Inc.). The measurement was carried out at an excitation wavelength of 492 nm, while the emission wavelength was set at 520 nm.

### 2.9. Seahorse XFe-96 Metabolic Flux Analysis

The Agilent Seahorse XFe96 analyzer was used to measure various metabolic parameters, including oxygen consumption rate (OCR), extracellular acidification rate (ECAR), total ATP production, and ATP rate index (Agilent Technologies Inc., Santa Clara, CA, USA). The experiment involved seeding 4 × 10^3^ CRC cells in a complete RPMI medium with 10% FBS in XFp FluxPak PDL 8-well plates (Agilent Technologies Inc.) for 24 h to promote adherence, followed by treatment with the desired treatment and timepoint. Before the experiment, the seahorse sensor cartridge was hydrated with sterile water and incubated in a non-CO_2_ incubator at 37 °C, with the seahorse calibrant replacing the water two hours before the experiment. On the day of the experiment, Seahorse XF RPMI (pH = 7.4) was supplemented with 1 mM pyruvate, 2 mM glutamine, and 10 mM glucose (Agilent Technologies Inc., Santa Clara, CA, USA). The complete RPMI medium was replaced with 180 μL of Seahorse XF base medium, and the cells were incubated in a non-CO_2_ incubator at 37 °C for 1 h. During this incubation period, oligomycin and rotenone/antimycin (Agilent Technologies Inc.,) were prepared for injection in the Seahorse XF RPMI medium to achieve final concentrations of 1.5 μM and 0.5 μM, respectively. To prepare for injection, 20 μL and 22 μL of oligomycin and rotenone/antimycin, respectively, were loaded into drug delivery ports A and B of the hydrated sensor cartridge. The cartridge was then loaded into the Seahorse XF analyzer (Agilent Technologies Inc.) for calibration for 30 min. Following calibration, the cell culture plate was loaded into the analyzer, and OCR and ECAR were monitored following the sequential injection of oligomycin and rotenone/antimycin. Finally, the ATP production rate in treated wells was compared to that of untreated wells according to the manufacturer’s instructions.

### 2.10. Western Blot Analysis

To investigate protein expression levels, a Western blot analysis technique was employed. For this purpose, CRC cells were initially seeded at a concentration of 1 × 10^6^ cells/mL in 60 mm Petri dishes. Following seeding, the cells were subjected to the previously detailed treatments and allowed to incubate for 24 h at 37 °C within a 5% CO_2_ incubator. After the designated treatment period, the CRC cells were harvested and lysed using RIPA buffer, which contained both protease and phosphatase inhibitors to preserve protein integrity (Sigma-Aldrich, St. Louis, MO, USA). Following protein quantification (Bio-Rad, Hercules, CA, USA), 30–40 µg of protein samples were loaded onto SDS-PAGE gels and subjected to electrophoresis to achieve separation. Subsequently, the separated proteins were transferred onto nitrocellulose membranes. To reduce non-specific binding, the membranes were blocked using a 5% non-fat milk solution in Tris-buffered saline with Tween 20 (TBST). The membranes were incubated overnight at 4 °C with primary antibodies against Cytochrome c (CYCS), cytochrome C oxidase subunit IV (COX IV), heat shock protein 60 (HSP60), prohibitin 1 (PHB1), pyruvate dehydrogenase (PDH), succinate dehydrogenase subunit A (SDHA), superoxide dismutase 1 (SOD1), voltage-dependent anion channel (VDAC) (CAT#8674, Cell Signaling Technology, Danvers, MA, USA), ERα and ERβ (Ab75635 and ab288, Abcam, Cambridge, UK), RPS2 and TMEM177 (NBP2-14852 and NBP2-31567, R&D Systems, Minneapolis, MN, USA), β-actin (CAT#4967s, Cell Signaling Technology, Danvers, MA, USA). Following this, the membranes were washed with TBST and subsequently incubated with secondary antibodies that corresponded to the primary antibodies used. All primary antibodies were used at a dilution of 1:1000, and secondary antibodies at 1:5000, following manufacturer recommendations. The images of the protein bands were captured using a chemiluminescence imaging system from (Bio-Rad, Hercules, CA, USA).

### 2.11. Mitochondrial Membrane Potential (MMP)

The CRC cells were seeded and allowed to adhere for 24 h in a CO_2_ incubator in a 96-well plate (Thermo Fisher Scientific). The MMP was measured using the JC-1-Mitochondrial Membrane Potential Assay kit (Abcam, Cat. No. ab113850). Briefly, following treatment, cells were washed once with 1X dilution buffer and then incubated with 10 µM JC-1 dye in 1X dilution buffer for 30 min at 37 °C, protected from light. JC-1 dye was then removed; the cells underwent two washes with 100 µL of 1X dilution buffer, and the final wash was retained without aspiration for reading purposes. The readings were measured using a Synergy™ HTX Multi-Mode microplate reader (Agilent Technologies Inc.) at excitation 535 nm and emission 590 nm. Pictures of the stained cells were taken using Olympus Fluorescence Microscope at 20X. The treatment readings were compared to the control reading after subtraction of the background fluorescence.

### 2.12. Quantitative Methylation PCR (qMSP)

To investigate the DNA methylation levels of *RPS2* and *TMEM177,* qMSP was employed because it provides high sensitivity for detecting promoter methylation changes in low-abundance cell populations and has been widely validated as an alternative to bisulfite sequencing in targeted functional studies. For this purpose, two sets of controls were created to represent fully methylated and fully unmethylated conditions, following the established methods [8]. The fully methylated control involved methylating genomic DNA with SssI methylase (New England Biolabs, Ipswich, MA, USA). On the other hand, the fully unmethylated control was generated by amplifying genomic DNA using the GenomiPhi amplification system (GE Healthcare, Chicago, IL, USA). An aliquot of genomic DNA (1 μg) was subjected to treatment with the EpiTect Bisulfite Kit (Qiagen, Hilden, Germany) to convert unmethylated cytosine to uracil while preserving methylated cytosines. Subsequently, qMSP was performed using 1 μL of the sodium bisulfite-treated DNA, along with primers specifically designed for methylated and unmethylated DNA (Supplementary Table S1), SYBR Green I fluorescent dye (for qMSP only; BioWhittaker Molecular Applications, Cambrex Bio Science, Copenhagen, Denmark), and an iCycler Thermal Cycler (Bio-Rad, Hercules, CA, USA), DNA methylation levels and the percentage of methylated reference were calculated based on the quantification cycle (Cq) values obtained from qMSP. qMSP was chosen to test functional association with transcriptional output. In this study, qMSP was integrated with ERα knockdown and pharmacologic demethylation to infer regulatory directionality.

### 2.13. Demethylation Treatment

Demethylation was performed according to a previously described protocol. Briefly, the CRC cell lines underwent demethylation treatment through three cycles, each lasting 24 h, using 5-Azacitidine (Aza) at a concentration of 1.0 µM [21].

### 2.14. Quantitative Real-Time Reverse Transcription (RT)-PCR

To initiate the process of cDNA synthesis, 1 µg of total RNA was utilized. The synthesis was carried out using the QuantiTect Reverse Transcription Kit from Qiagen, following the manufacturer’s guidelines. Following the cDNA synthesis, RT-PCR was executed using 1 µL of the synthesized complementary DNA (cDNA) along with specific primers that were designed for the targeted genes (Supplementary Table S2). The Qiagen Rotor-gene Q-PCR system from Qiagen was utilized for this purpose. In the analysis, the expression levels of the intended human genes were normalized to the expression of GAPDH.

### 2.15. Bioinformatics Analysis

The evaluation of variations in gene expression and promoter methylation level between neighboring healthy tissues and tumor tissues in CRC was conducted through the University of Alabama at Birmingham Cancer data analysis Portal (UALCAN) database. We also utilized the publicly accessible University of California Santa Cruz Genome Browser (http://genome.ucsc.edu/, accessed on 25 September 2023) to investigate the presence of CpG islands (CGI) within the promoter regions of candidate genes. The UALCAN database was also utilized to identify genes that were negatively correlated with *ESR1* expression in CRC. To enhance transparency, we applied a stepwise filtering pipeline: (1) Genes negatively correlated with *ESR1* (*n* = 169); (2) Genes annotated to OXPHOS-related pathways (*n* = 56); (3) Genes significantly upregulated in CRC clinical tissue (*n* = 19); (4) Genes containing promoter CGIs (*n* = 12); (5) Genes displaying consistent hypomethylation in CRC tissue (*n* = 2: *RPS2* and *TMEM177*). Only *RPS2* and *TMEM177* passed all filters, explaining why these candidates preferentially emerged as epigenetically regulated OXPHOS-linked genes. An enrichment analysis of these genes was performed using the Metascape database (https://metascape.org/, accessed on 25 September 2023). We also used another database, ShinyGO v0.741 database, to confirm our findings (http://bioinformatics.sdstate.edu/go74/, accessed on 25 September 2023). Additionally, the Gene Expression Profiling Interactive Analysis, version 2 tool (GEPIA2) database (http://gepia2.cancα-pku.cn/#index/, accessed on 25 September 2023) was employed to analyze the correlation between candidate genes mRNA expression in CRC. All results with a log-rank *p*-value of <0.05 were considered significant.

### 2.16. Statistical Analysis

All experiments were independently repeated three times (*n* = 3 biological replicates) unless otherwise specified. Data are presented as mean ± SEM, as indicated in all figure legends. Statistical significance was determined using Student’s *t*-test for two-group comparisons or one-way ANOVA followed by Tukey’s post hoc test for multiple comparisons. Because our functional assays involved hypothesis-driven comparisons rather than high-dimensional screening, formal power analysis was not required; however, triplicate independent experiments provided adequate reproducibility, and all statistically significant findings were consistently observed across replicates. A threshold of *p* < 0.05 was considered significant. For additional statistical computations, Microsoft Excel 2010 was utilized. To quantify the Western blot bands and provide a visual representation of the results, ImageJ software (version 1.54) was employed. A representative figure was crafted to illustrate the quantified bands from the Western blot analysis. All blots were normalized to β-actin, and corresponding loading controls were included for each experimental set.

## 3. Results

### 3.1. E2 Treatment Reduces CRC Cell Survival In Vitro

**Figure 1:**
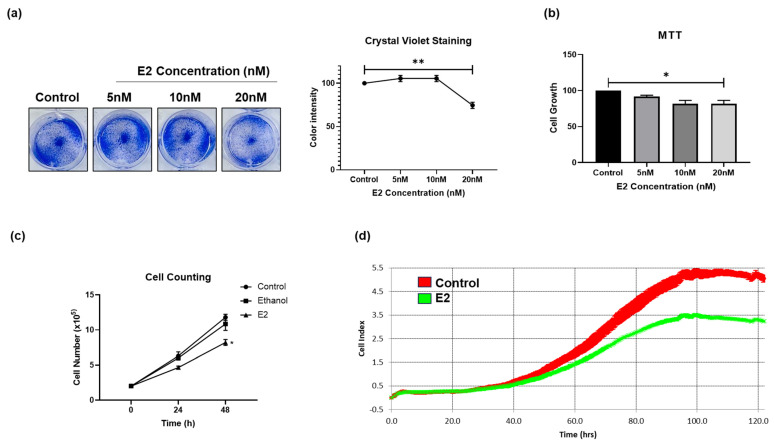
Effect of E2 treatment on colorectal cancer (CRC) cell survival. (**a**) HCT-116 cells were stained with crystal violet (CV) to measure cell proliferation following treatment with increasing concentrations of E2 for 48 h. (**b**) HCT-116 cell survival was measured using (3-4,5-dimethyl-2-thiazolyl)-2,5-diphenyl-2-H-tetrazolium bromide (MTT) following treatment with increasing concentrations of E2 for 48 h. (**c**) HCT-116 cells were stained using trypan blue and counted under the microscope following ethanol, vehicle, and E2 treatment for 24 and 48 h. (**d**) Real-Time Cell Analysis (RTCA) was used to measure real-time HCT-116 cell proliferation for up to 120 h. Data are shown as mean ± SEM from *n* = 3 independent experiments. Statistical analysis was performed using one-way ANOVA followed by Tukey’s test unless otherwise indicated. * *p*-value < 0.05, and ** *p*-value < 0.01.

To assess the potential cytotoxic impact of E2 treatment on the growth and survival of CRC cells, two CRC cell lines, HCT-116 and SW480 were treated with increasing concentrations of E2 for 48 h. The results show that CV stain intensity was reduced from 100% to 70% in HCT-116 cells treated with E2 (Figure 1a, *p* < 0.01. Statistical significance for all quantitative assays in Figure 1 was assessed using one-way ANOVA with Tukey’s post hoc test). Cell viability was reduced at 20 nM E2 in HCT-116 cells by 20% relative to the control (Figure 1b, *p* < 0.05). Additionally, although the cell counts in E2-treated HCT-116 cells increased (from 2 × 10^5^ to 8.2 × 10^5^ cells/well) within 48 h, it was significantly lower than that in untreated cells (2 × 10^5^ to 11.8 × 10^5^ cells/well) or cells treated with vehicle (Figure 1c, *p* < 0.05). Similar results were observed in SW480 (Supplementary Figure S1a–c). Although E2 markedly reduced cell proliferation, we did not observe morphological indicators typically associated with distinct cell-cycle arrest phases (e.g., enlarged G2/M-phase morphology or flattened G1-arrested phenotypes). The reduction in real-time proliferation index and metabolic output suggests that E2 primarily induces a metabolic-dependent proliferative slowdown, which is consistent with literature describing energy-restriction-mediated cytostasis independent of classical checkpoint arrest. We further confirmed the effect of E2 on CRC cells using RTCA by measuring cell-induced electrical impedance values. This approach allowed for the quantification of the kinetics of the cellular response in real time. Higher CI values represent increased cell viability, whereas lower CI values represent decreased cell viability. E2 treatment significantly reduced CI value in HCT-116 cells (from 5 to 3.2) (Figure 1d). Taken together, these findings suggest that E2 may reduce CRC carcinogenesis by attenuating their survival and inhibiting proliferation.

### 3.2. E2 Treatment Alters CRC Cell Bioenergetics

**Figure 2:**
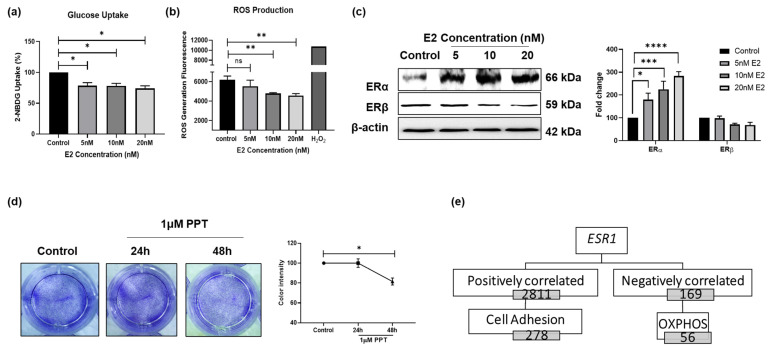
E2-ERα signaling modulates glucose metabolism. (**a**) 2-[N-(7-nitrobenz-2-oxa-1,3-diazol-4-yl) amino]-2-deoxy-glucose (2-NBDG) uptake level was measured using flow cytometry in HCT-116 cells treated with increasing concentrations of E2 for 48 h. (**b**) Reactive Oxygen Species (ROS) generation was measured by the 2′,7′-Dichlorofluorescin diacetate (DCFDA) assay in HCT-116 cells treated with increasing concentrations of E2 for 48 h. (**c**) Estrogen receptor α (ERα) and estrogen receptor β (ERβ) expression was assessed using a Western blot with E2 treatment in HCT-116 cells. (**d**) The effect of the propyl pyrazole triol (PPT), selective ERα agonist, 24 and 48 h treatment on HCT-116 proliferation. (**e**) A flowchart representing genes positively or negatively correlated with *ESR1* gene expression in CRC patients. Data are shown as mean ± SEM from *n* = 3 independent experiments. Statistical analysis was performed using one-way ANOVA followed by Tukey’s test unless otherwise indicated. ns Not significant, * *p*-value < 0.05, ** *p*-value < 0.01, *** *p*-value < 0.001 and **** *p*-value < 0.0001.

To explore the mechanism(s) through which E2 reduces CRC cell survival, its capacity to reprogram CRC cell energy metabolism was examined. As shown in Figure 2, 48 h E2 treatment resulted in a substantial reduction in glucose uptake and ROS production in both HCT-116 and SW480 cells (Figure 2a,b and Figure S1d,e). As E2 exerts its major effects through its binding to ERα or ERβ, we next assessed the expression of these two receptors on CRC. While ERβ showed no significant change following E2 treatment, the expression of ERα significantly increased in E2-treated HCT-116 cells (Figure 2c). Basal ERα expression was detectable in both HCT116 and SW480 cells, whereas ERβ levels were low and unchanged by E2 treatment (Figure 2c), supporting the use of these lines as ERα-responsive CRC models. ERβ expression did not significantly change under our treatment conditions, and PPT (ERα-selective agonist) reproduced key phenotypes, supporting ERα-dominant signaling in this experimental setting. Accordingly, subsequent mechanistic analyses focused on ERα. Additionally, treatment of HCT-116 cells with PPT, a selective ERα agonist, for 24 and 48 h resulted in a significant reduction in cell proliferation as measured by CV staining and RTCA CI (Figure 2d and Figure S2a). Notably, *ESR1* mRNA expression was found to be significantly downregulated in CRC tissue when compared to normal tissue (Supplementary Figure S2b).

Based on these findings, genes with positive and negative correlations with *ESR1* expression in CRC tissues were identified (Supplementary Table S3). Pathway enrichment analysis of genes positively (2811) and negatively (169) associated with *ESR1* expression in CRC patients (Figure 2e) was performed. As E2 showed a protective effect in CRC cells, the focus was on genes that negatively correlated with *ESR1* expression. We prioritized metabolic/OXPHOS-related genes within the ESR1-negatively correlated set because our functional data (glucose uptake, OCR/ECAR, ATP production, ROS, and MMP) indicated that E2-ERα signaling predominantly reprograms CRC bioenergetics. Thus, focusing on OXPHOS-linked candidates provided a hypothesis-driven bridge between the observed phenotype (mitochondrial suppression) and candidate epigenetic targets. Pathway enrichment analysis revealed that 169 identified genes are enriched in pathways related to cellular metabolism including the electron transport chain (ETC), OXPHOS pathway (WP111), mitochondrial translation initiation (R-HSA-5368286), and mitochondrial organization (GO:0007005). These analyses consistently highlighted OXPHOS/ETC-related enrichment among ESR1-negatively correlated genes. (Supplementary Figure S2c and Table S4). These findings were further validated using additional databases, which showed enrichment of pathways related to OXPHOS, ATP synthesis, and ETC (Supplementary Figure S2d). Therefore, we suggest that E2 might reduce CRC cell survival through its receptor ERα in which the E2/ER*α* complex may attenuate their energy production metabolism.

### 3.3. E2 Effect on CRC Growth and Metabolism Is ERα-Dependent

**Figure 3:**
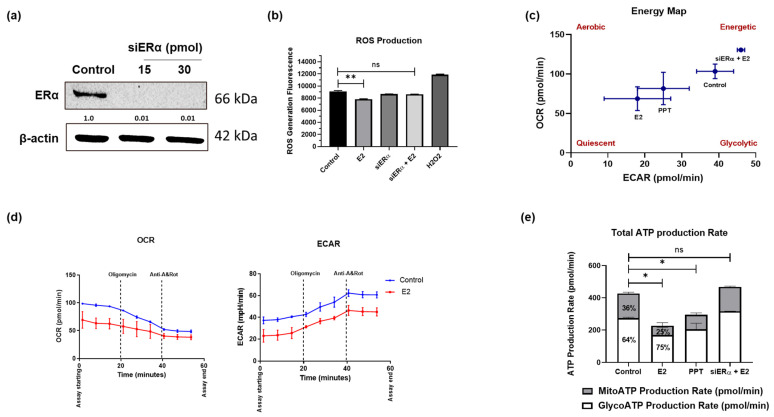
ERα silencing diminishes the effect of E2 on ATP production in HCT-116 cells. (**a**) ERα knockdown was confirmed using a Western blot. Representative blots are shown; corresponding β-actin loading controls and densitometry (normalized to β-actin) are provided. (**b**) Cellular ROS generation was measured by DCFDA assay following treatment with 20 nM E2 with/without siERα in HCT-116 cells. (**c**) An energy map was used to represent general Seahorse XF ATP rate assay results following treatment with 20 nM E2 with/without siERα in HCT-116 cells. (**d**) The kinetic profile of oxygen consumption (OCR) and extracellular acidification (ECAR) was measured using Seahorse XF ATP rate assay following E2 treatment. (**e**) Net ATP production rate from mitochondrial and glycolytic ATP production following E2, PPT, and siERα treatments. Data are shown as mean ± SEM from *n* = 3 independent experiments. Statistical analysis was performed using one-way ANOVA followed by Tukey’s test unless otherwise indicated. ns Not significant, * *p*-value < 0.05, and ** *p*-value < 0.01.

To validate the role of ERα in E2 signaling in CRC cells, we knocked down its expression in combination with E2 treatment (Figure 3a). Notably, we found that silencing ER*α* expression in HCT-116 cells significantly reduced the ability of E2 to reduce ROS production (Figure 3b). To ascertain whether silencing ER*α* could alter ATP production we used the Seahorse XF ATP rate assay. The energy map indicated a shift in HCT-116 cell metabolism towards a quiescent energetic form in the presence of either E2 or PPT; E2 treatment with ERα knockdown did not affect cellular metabolism (Figure 3c). Both OCR and ECAR rates were downregulated with E2 treatment (Figure 3d). Mitochondrial, glycolytic, and total ATP were all also reduced in cells treated with E2 or PPT. Interestingly, the ability of E2 treatment to reduce ATP production was diminished in E2-treated ERα-silenced cells (Figure 3e). Interestingly, we found that control HCT-116 cells depend on mitochondrial ATP by 36% while 64% of their energy is generated from glycolytic ATP. However, in E2-treated HCT-116 cells, 25% of total ATP came from mitochondrial ATP while 75% was from glycolytic ATP. More reduction in mitochondrial ATP (from 152 pmol/min to 57.5 pmol/min) than in glycolytic ATP (from 273 pmol/min to 170 pmol/min) was observed in E2-treated relative to control cells. Collectively, our findings suggest that the E2-ERα complex may attenuate ATP production in CRC cells by inhibiting mitochondrial ATP production and the OXPHOS pathway.

### 3.4. E2-ERα Complex Induces Mitochondrial Membrane Depolarization and Disrupts the OXPHOS Pathway

**Figure 4:**
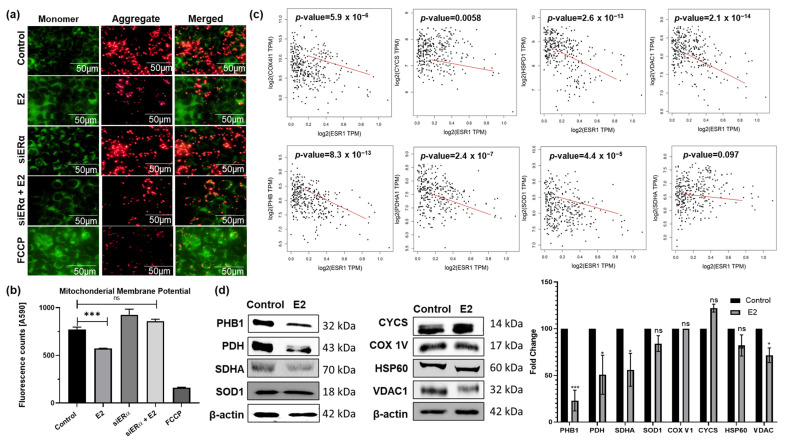
E2 signaling modulates mitochondrial function in HCT-116 cells. (**a**) The depolarization of MMP was examined at 20X magnification following treatment of ERα-silenced HCT-116 cells with 20 nM E2. Increased mitochondrial permeability is indicated by red color and dye accumulation in the cytoplasm is indicated by green color. (**b**) Quantitative analysis of MMP at 590 nm. (**c**) Correlation between *ESR1 * mRNA expression and eight mitochondrial markers in CRC patients. Red line is the line of best fit (Regression Line) (**d**) Protein expression of mitochondrial markers in E2-treated HCT-116 cells by Western blotting. Representative blots are shown; corresponding β-actin loading controls and densitometry as bar graph (normalized to β-actin) are provided. Data are shown as mean ± SEM from *n* = 3 independent experiments. Statistical analysis was performed using one-way ANOVA followed by Tukey’s test unless otherwise indicated. ns Not significant, * *p*-value < 0.05, and *** *p*-value < 0.001.

To explore how E2 inhibits mitochondrial ATP production, we stained CRC cells with JC-1. While high MMP leads to the formation of red fluorescent JC-1 aggregates, low MMP results in diffuse green fluorescence. E2 treatment resulted in a significant mitochondrial membrane depolarization in HCT-116 cells, and this effect was inhibited with the knockdown of ERα (Figure 4a,b). Comparable results were observed in SW480 cells (Supplementary Figure S3a,b). These findings were validated in control CRC cells treated with the uncoupling agent carbonylcyanide-p-trifluoromethoxyphenylhydrazone (FCCP), which resulted in the loss of MMP and the development of diffuse green fluorescence. The expression of eight different mitochondrial markers revealed a significant negative correlation with *ESR1* in CRC patients (Figure 4c). The expression of mitochondrial markers PHB1, PDH, SDHA, and VDAC was reduced in such cells (Figure 4d). Thus, we propose that E2 interferes with mitochondrial function, consequently inhibiting OXPHOS in CRC cells. Reduced ROS likely reflects suppressed electron transport activity rather than enhanced antioxidant protection.

### 3.5. RPS2 and TMEM177 Are Epigenetically Regulated by E2 in CRC Cells

**Figure 5:**
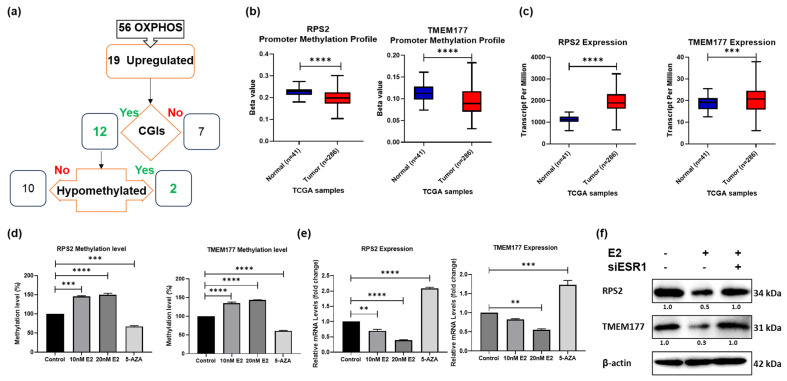
Identification of potential epigenetic targets for E2 in CRC. (**a**) A flowchart representing genes with the potential to be targeted by E2 signaling in CRC cells. (**b**) *RPS2* and *TMEM177* promoter methylation profile in CRC versus normal tissue. (**c**) *RPS2* and *TMEM177* mRNA expression in CRC versus normal tissue. (**d**) *RPS2* and *TMEM177* methylation levels following 10 and 20 nM E2 or 5-Aza-2-deoxycytidine (Aza) treatment in HCT-116 cells. (**e**) *RPS2* and *TMEM177* mRNA expression following 10 and 20 nM E2 or Aza treatment in HCT-116 cells. (**f**) *RPS2* and *TMEM177* protein expression following 20 nM E2 with or without siESR1 treatment in HCT-116 cells. Representative blots are shown; corresponding β-actin loading controls and densitometry (normalized to β-actin) are provided. Data are shown as mean ± SEM from *n* = 3 independent experiments. Statistical analysis was performed using one-way ANOVA followed by Tukey’s test unless otherwise indicated. ** *p*-value < 0.01, *** *p*-value < 0.001 and **** *p*-value < 0.0001.

To identify potential targets for the E2-ERα complex in CRC, we screened 169 genes that negatively correlated with ERα expression in clinical samples of CRC. By only selecting genes specifically associated with the OXPHOS pathway, the list of genes was narrowed to 56 and by only selecting those that were upregulated in CRC clinical samples further narrowed the list to 19 genes. By focusing on genes potentially subject to DNA methylation owing to the presence of CGI in their promoters, although 12 genes contained CGIs, only *RPS2* and *TMEM177* met the combined criteria of OXPHOS involvement + CRC overexpression + consistent hypomethylation, which justified their selection as the most biologically robust candidates. Both genes showed concordant CRC-associated hypomethylation with relative overexpression in patient datasets. (Figure 5a, Figures S4 and S5b,c). To assess whether the suppressive impact of E2 on RPS2 and TMEM177 gene expression is promoter hypermethylation-related, qMSP analysis of extracts isolated from E2-treated HCT-116 and SW480 cells was performed. Treatment with E2 at 10 and 20 nM induced a significant increase in *RPS2* and *TMEM177* promoter methylation. Conversely, when treated with the demethylating agent Aza, a reversal of the inhibitory effects of E2 on the expression of *RPS2* and *TMEM177* in both cell lines was observed (Figure 5d and Figure S4b). Moreover, treating both cell lines with 10 and 20 nM E2 reduced the mRNA expression of both genes while their expression was increased when treated with Aza (Figure 5e and Figure S4c). Furthermore, both RPS2 and TMEM177 protein expression was downregulated in E2-treated cells; however, this downregulation was not detected in ESR1-silenced cells (Figure 5f). Protein-level suppression by E2 was lost upon ERα knockdown, supporting ERα dependence (Supplementary Figure S4d). This indicates that gene promoter methylation is a critical factor in mediating the influence of E2 in CRC cells. These findings are consistent with ERα-associated recruitment of chromatin-modifying machinery reported in other cancer systems, whereby ligand-activated ERα influences DNMT activity indirectly through signaling intermediates rather than direct promoter binding, which was not directly tested here.

### 3.6. RPS2 and TMEM177 Silencing Induces Apoptosis and Inhibits Mitochondrial Activity in HCT-116 Cells

**Figure 6:**
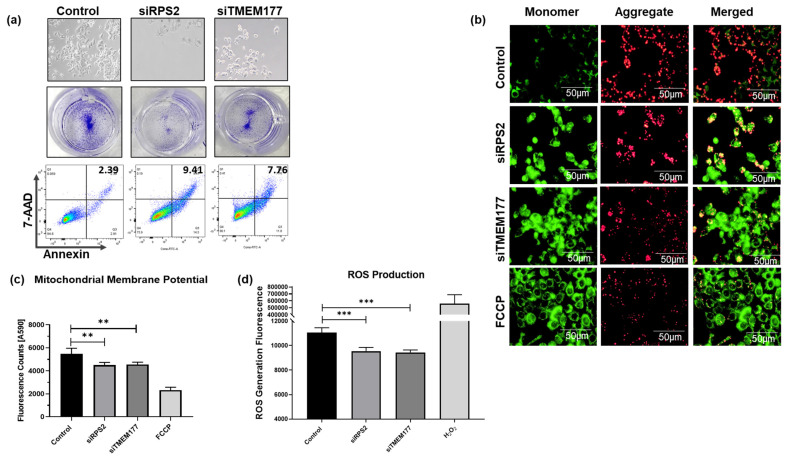
*RPS2* and *TMEM177* enhance cell viability and sustain mitochondrial function in HCT-116 cells. (**a**) Cell morphology, CV, and apoptosis were evaluated in HCT-116 cells treated with 30 pmol of siRPS2 or siTMEM177. (**b**) Depolarization of MMP was observed after silencing *RPS2* and *TMEM177* in HCT-116 cells, 20X magnification. Increased mitochondrial permeability as indicated by red color, green color indicates cytoplasmic dye accumulation (**c**) Quantitative analysis of the MMP at 590 nm. (**d**) ROS generation in *RPS2-* and *TMEM177*-silenced HCT-116 cells as measured by the DCFDA assay. Data are shown as mean ± SEM from *n* = 3 independent experiments. Statistical analysis was performed using one-way ANOVA followed by Tukey’s test unless otherwise indicated. ** *p*-value < 0.01 and *** *p*-value < 0.001.

To assess the impact of E2-induced suppression of *RPS2* and *TMEM177* gene expression in HCT-116 cells, siRNA silencing was conducted using 15 and 30 pmol of siRNAs targeting these genes (Supplementary Figure S5a,b). *RPS2*- or *TMEM177*-silenced HCT-116 cells experienced alterations in cell morphology characterized by cell shrinkage and irregular shape along with a noticeable decrease in cell survival and cell proliferation as measured by CV staining and the MTT assay, especially at 30 pmol siRNA (Figure 6a) (Supplementary Figure S5c,d). Significant levels of apoptosis were also observed in *RPS2*- and *TMEM177*-silenced cells relative to controls (Figure 6a and Figure S5e). As Aza can exert genome-wide demethylation, we interpret Aza-based rescue as supportive (not standalone) evidence, and we emphasize that the key specificity is provided by the ERα dependence and qMSP-detected promoter methylation shifts for RPS2 and TMEM177. Lastly, ROS levels and MMP following the knockdown of *RPS2* and *TMEM177* were reduced (Figure 6b–d). Although DCFDA is a general ROS indicator, the marked decrease we observed is consistent with reduced mitochondrial OXPHOS activity—the primary cellular ROS generator. Because mitochondrial depolarization was independently confirmed (Figure 4), the drop in ROS is mechanistically attributable to reduced electron-transport flux. Taken together, these observations suggest that RPS2 and TMEM177 are critically required for the proper ATP production and survival of CRC cells. Although RPS2 is classically known as a ribosomal protein, emerging evidence shows that ribosomal proteins regulate mitochondrial biogenesis, metabolic stress responses, and translational programs affecting OXPHOS-related enzymes. TMEM177, recently implicated in COX2 biogenesis within Complex IV, directly links it to mitochondrial respiratory chain assembly, explaining why its suppression disrupts OXPHOS phenotypes. Complex IV (cytochrome c oxidase) is a key control point in electron transport and maintenance of mitochondrial membrane potential. Disruption of Complex IV biogenesis can reduce electron flow, destabilize MMP, limit ATP synthesis, and shift redox balance, collectively impairing survival of cancer cells that retain mitochondrial dependency. Therefore, TMEM177 suppression provides a plausible mechanistic route to OXPHOS attenuation and viability loss observed in our CRC models. Therefore, the epigenetic silencing induced by E2 on these two genes may represent a mechanistic axis that suppresses CRC cell viability in vitro.

## 4. Discussion

E2 signaling has been previously shown to modulate various aspects of mitochondrial function, ranging from ATP production to ROS generation, MMP, and calcium metabolism [22]. That said, the ability of E2 signaling to regulate ATP production and mitochondrial function in CRC cells has not been fully explored. Data presented here demonstrate for the first time that engagement of E2 with its cognate ERα receptor exerts anti-proliferative effects in our CRC models by disrupting MMP and depriving cells of ATP. Two novel E2 target genes, *RPS2* and *TMEM177*—both highly expressed in CRC—were found to negatively correlate with *ESR1* mRNA expression and their promoter regions were also hypomethylated in human CRC tissue samples. Our clinical inferences are derived from multiple independent public CRC cohorts (expression, methylation, and correlation analyses), providing strong patient-scale support. Nonetheless, direct validation in an independent local tissue cohort would further strengthen translational generalizability and represent a logical next step. The stringent multi-layer filtering explains why only two genes met all criteria; the modest effect sizes observed in TCGA datasets reflect typical methylation-regulated transcriptional modulation, where relatively small expression changes can generate disproportionately large functional effects in metabolic pathways. E2 treatment resulted in hypermethylation and suppression of *RPS2* and *TMEM177* expression in CRC cells. Knockdown these genes produced anti-cancer effects that closely paralleled those observed following E2 treatment.

Metabolic reprogramming, characterized by significant alterations in cellular energy utilization and nutrient metabolism, is a recognized hallmark of cancer and plays a central role in cancer progression [14]. In many cancer types, glycolysis represents the predominant source of ATP; however, certain cancers retain reliance on OXPHOS for energy generation [22]. In addition, specific tricarboxylic acid (TCA) cycle intermediates are elevated and contribute to cancer progression [16]. Both HCT116 and SW480 are Kirsten rat sarcoma virus (*KRAS*)-mutant CRC cell lines [19], and increasing evidence indicates that *KRAS* mutations facilitate metabolic reprogramming in CRC and other cancers [23]. The role of OXPHOS in CRC remains controversial, with some studies reporting its downregulation and supporting its continued activity and contribution to ATP production [24]. Our results demonstrate that CRC cells exhibit elevated mitochondrial activity and OXPHOS pathway engagement, as evidenced by the increased OCR and mitochondrial ATP production, which accounted for more than one-third of total cellular ATP. These findings suggest that targeting OXPHOS represents a promising avenue for CRC prevention and therapy. While flow cytometric cell-cycle profiling can delineate checkpoint-specific arrest, our findings indicate that proliferative inhibition is closely linked to mitochondrial depolarization and ATP depletion—an established mechanism of metabolic cytostasis that can occur independently of overt G1/S or G2/M arrest. Thus, E2-induced growth suppression in CRC cells is best explained by energy deprivation–driven cytostasis rather than checkpoint-specific cell-cycle arrest, consistent with the observed mitochondrial dysfunction.

The relationship between mitochondrial activity, OXPHOS, and E2 remains an active area investigation. While many aspects are still unclear, several studies have demonstrated that E2 influences mitochondrial morphology and gene expression [25,26,27,28]. Moreover, E2 plays a regulatory role in ROS production and helps maintain mitochondrial integrity in normal cells [29,30]. In cancer cells, however, E2 appears to exert dual effects, either preserving or inducing mitochondrial depolarization depending on cellular context [16,31]. For example, in neuroblastoma cells, E2 preserves mitochondrial ATP production through enhanced OXPHOS [31], whereas, in cervical cancer, E2 induces mitochondrial dysfunction and suppresses expression of multiple OXPHOS pathway genes [16]. Consistent with these observations, our findings demonstrate that E2 disrupts ATP production, induces mitochondrial depolarization, suppresses the OXPHOS pathway, and reduces CRC cell survival.

We also observed a reduction in ROS levels, consistent with diminished OXPHOS activity. Mitochondria are a major source of cellular ROS, and cancer cells typically maintain elevated baseline ROS levels due to an imbalance between ROS generation and antioxidant capacity [32]. ROS play diverse roles in cellular signaling, influencing proliferation, survival, differentiation, and invasion [22]. Although excessive ROS can induce cellular damage, cancer cells often require a permissive redox tone to sustain signaling and bioenergetic flux. Thus, reduced ROS reflects impaired mitochondrial function and reduced energy output, which can limit proliferation and promote apoptosis. In this context, ROS reduction is a consequence of OXPHOS suppression rather than survival-enhancing antioxidant effect.

E2 has also been shown to enhance antioxidant mechanisms, counteracting oxidative stress by inducing expression of antioxidant proteins such as glutathione, thioredoxin, and superoxide dismutase [16]. Although additional metabolic flux assays could add granularity, the concordant findings of mitochondrial depolarization, reduced OCR/ATP output, and apoptosis sufficiently support impaired OXPHOS in this setting. While our data supports the role of RPS2 and TMEM177 in maintaining mitochondrial integrity and OXPHOS output, the precise molecular mechanisms were not dissected. Importantly, loss of MMP represents a proximate functional marker of impaired electron transport coupling, supporting the proposed axis at a systems level. Recent studies have further demonstrated that E2 influences the expression of key proteins involved in mitochondrial function. Specifically, E2 inhibits the activity of mitochondrial complexes I, II, III, and IV, all of which are essential for ATP production via OXPHOS [25]. E2 also affects mitochondrial ATP synthase (F0F1-ATPase), which catalyzes ATP synthesis from ADP and inorganic phosphate using the energy generated by the ETC [25]. While mitochondria-specific probes (e.g., MitoSOX) could further localize ROS changes, the combined suppression of OCR/mitochondrial ATP and collapse of membrane potential strongly supports reduced mitochondrial electron transport–derived ROS.

Our findings further demonstrate that E2 treatment reduces protein expression of four mitochondrial markers: PHB1, PDH, SDHA, and VDAC. These proteins are known to be upregulated in cancer and to contribute to tumor progression [33,34,35,36]. Within mitochondria, PHBs predominantly exist as membrane-bound ring complexes and act as chaperones that preserve mitochondrial protein stability [37]. The PDH complex, located in the mitochondrial matrix, converts pyruvate to acetyl-CoA by decarboxylation [38]. SDHA, also known as Complex II or succinate quinone oxidoreductase, plays a central role in both the TCA and ETC [39]. VDAC is ubiquitously expressed in the outer mitochondrial membrane and serves as a major conduit for metabolite exchange mitochondria and the cytosol [40]. Collectively, these proteins may represent potential therapeutic targets in CRC, although further investigation is required.

Two CRC cell lines—HCT116 and SW480—were employed in this study. Examining E2 effects in both cell lines, which represent early- and late-stage adenocarcinoma, is critical for understanding the diverse roles of E2 in CRC progression. Early-stage SW480 cells allow assessment of E2 effects on tumor initiation and early growth, whereas late-stage HCT116 cells provide insight into advanced tumor behaviors such as resistance to apoptosis. Studying these distinct stages improves understanding of potential stage-specific therapeutic implications of E2 in CRC [18]. Notably, E2 effects were more pronounced in HCT116 cells than in SW480 cells. This heightened may reflect advanced tumor stage, accumulation of additional mutations, or altered E2 metabolic signaling networks. Moreover, the stronger response in HCT116 relative to SW480 is consistent with differences in ERα responsiveness and baseline metabolic wiring between CRC lines. Importantly, core mechanistic signatures—including mitochondrial depolarization, reduced gene expression, and methylation shifts—were observed in both models, supporting generalizability within ERα-expressing CRC contexts.

Identifying regulators of gene expression during cancer progression remains a major challenge in oncology. Epigenetic alterations, particularly DNA methylation, play critical role in regulating genes involved in metabolic reprogramming and ATP production in CRC [14]. DNA methylation and demethylation frequently affect tumor suppressor genes and oncogenes enriched in CGIs [41]. The role of E2 in epigenetic regulation and DNA methylation has been recognized previously [8,9]; however, to our knowledge, this is the first study demonstrating that E2-induced DNA hypermethylation regulates metabolic reprogramming and the OXPHOS pathway in CRC. Previous studies have linked epigenetic regulation of OXPHOS genes to cancers, Such as hypermethylation-mediated suppression of Cytochrome C Oxidase Subunit 7A1 (COX7A1) in breast cancer [42]. Here, we demonstrate that E2 induces hypermethylation and downregulation of two novel metabolic genes, *RPS2* and *TMEM177*, in CRC. Although bisulfite sequencing offers base-resolution methylation analysis, qMSP reliably quantifies promoter methylation changes and is sufficient when the objective is to link methylation with functional outcome. Combined ERα dependence, Aza-mediated reversal, and transcriptional changes provide strong convergent evidence of promoter-level epigenetic regulation. While ChIP could confirm direct ERα recruitment, the observed ERα dependence of methylation changes and transcriptional repression is consistent with an ERα-linked mechanism (direct or indirect) without necessitating ChIP validation at this stage. We did not perform ERα ChIP-qPCR in this study; therefore, direct ERα occupancy at the RPS2/TMEM177 promoter regions remains hypothetical, and we interpret the methylation changes as ERα-dependent but potentially indirect. Our study prioritizes functional causality over molecular cataloging, demonstrating that ERα activation precedes promoter hypermethylation, transcriptional repression, and metabolic collapse—criteria sufficient to establish ERα-dependent epigenetic regulation.

RPS2 plays a role in assembly of the small 40S ribosomal subunit [43], and defects in ribosomal proteins have been linked to metabolic reprogramming in cancer cells [44,45]. Regarding TMEM177, no direct studies have implicated this protein in cancer progression. However, TMEM177 is involved in Cyclo-oxygenase 2 (COX2) biogenesis, a critical component of Complex IV in the mitochondrial respiratory chain [46]. Thus, although neither gene has been widely studied in cancer metabolism, their functional attributes align with OXPHOS regulation: RPS2 influences mitochondrial translation fidelity and biogenesis, whereas TMEM177 participates in Complex IV assembly. The pronounces mitochondrial phenotypes observed following their silencing are therefore mechanistically plausible despite modest expression differences in TCGA datasets. Moreover, because maintenance of mitochondrial membrane potential is essential for oxidative phosphorylation, its collapse following *RPS2* or *TMEM177* silencing functionally confirms impaired OXPHOS activity.

ERβ has been reported as a major estrogen receptor in colonic epithelium and may exert context-dependent effects in CRC. In our models, ERβ expression was not modulated by E2, and ERα-selective activation using PPT phenocopied E2 effects, indicating ERα predominance in mitochondrial-epigenetic axis described here. Future studies may examine whether ERβ modulates these outcomes under alternative receptor-expression contexts. The mechanism linking ERα activation to promoter hypermethylation is not necessarily a direct DNA-binding event. Rather, the ERα-dependent hypermethylation observed in this study is consistent with prior reports demonstrating indirect modulation of DNMT1 and DNMT3A/3B activity via kinase-mediated signaling and transcriptional co-regulators [47]. Accordingly, methylation changes likely occur downstream of ERα-dependent signaling cascades and precede reductions in gene expression. Because methylation changes were detectable at 24–48 h—an interval consistent with DNMT-mediated modification—it is plausible that methylation act as an upstream driver rather than a secondary consequence. The coherence between ERα knockdown, methylation suppression, and Aza rescue further supports this causal sequence. Future studies measuring DNMT dynamics and ERα promoter occupancy will help distinguish direct from indirect regulation; here, we provide functional ERα-dependence with promoter methylation and transcriptional readouts.

Mechanistically, ER signaling has been reported to influence DNA methylation through both direct and indirect routes, including modulation of DNMT expression and activity as well as recruitment of chromatin co-regulators. In many contexts, ERα exerts these effects via downstream signaling cascades and co-factor networks rather than through direct promoter binding [48,49]. Consistent with this literature, our findings support an ERα-linked DNMT-associated mechanism, although delineating the precise intermediate steps will require future investigation.

From a translational perspective, ERα-selective modulation (e.g., ERα agonism versus antagonism depending on tumor receptor context) may represent a strategy to suppress OXPHOS-supported CRC survival programs. Conversely, DNMT inhibitors could counteract promoter hypermethylation and restore RPS2/TMEM177 expression, with outcomes likely dependent on tumor metabolic dependency. These findings highlight the importance of biomarker-guided stratification based on ERα status and metabolic phenotype prior to considering endocrine-epigenetic combination strategies. With respect to translational relevance, circulating estradiol levels—particularly in postmenopausal settings—are generally below the upper range used in vitro. Nevertheless, tissue-level exposure can differ from plasma measurements, and mechanistic studies commonly apply concentrations that ensure robust ERα activation. Importantly, our findings establish an ERα-dependent mechanism; future work can determine minimal effective concentrations and whether pharmacologic ER modulators under clinically relevant hormonal controls.

While this study provides important insights into the role of E2 in CRC, certain limitations should be acknowledged. Restriction to two cell lines may limit the generalizability, as these models cannot fully capture tumor heterogeneity. However, these cell lines represent distinct disease stages and genetic backgrounds. Future work will extend these findings to additional CRC models, including patient-derived systems and in vivo approaches, to better reflect inter-patient variability and tumor microenvironmental influences. Although overexpression-based rescue studies can reinforce linear causality, such approaches may bypass endogenous epigenetic regulation and introduce supraphysiological expression artifacts. Our strategy—linking ERα-dependent hypermethylation, gene suppression, and phenocopy of E2-mediated mitochondrial dysfunction through gene silencing—provides strong mechanistic coherence without reliance on overexpression.

## 5. Conclusions

In conclusion, our findings indicate that E2 signaling suppresses CRC cell survival by disrupting key cellular processes, including the OXPHOS metabolic pathway. This work identifies previously unrecognized α-dependent epigenetic mechanism through which E2 hypermethylates and silences *RPS2* and *TMEM177*, thereby impairing mitochondrial function and reduce cellular energy output. These results open new avenues for therapeutic intervention targeting estrogen signaling and metabolic vulnerabilities in CRC. Because Aza has global epigenetic effects, it was used here as a mechanistic probe in conjunction with promoter-specific methylation analyses and ERα knockdown, enhancing specificity for the proposed ERα–methylation–transcription axis. Finally, given the context-dependent nature of E2 signaling, our conclusions are restricted to ERα-expressing CRC cell models under the experimental conditions used. Future studies evaluating selective estrogen receptor modulators, ERα-targeted agents, and epigenetic therapies may further clarify the therapeutic potential of this regulatory axis.

## Data Availability

The authors confirm that the data supporting the findings of this study are available within the article and its Supplementary Materials.

## Supplementary Materials

The following supporting information can be downloaded at: https://www.mdpi.com/article/10.3390/cells15020124/s1, Figure S1: Effect of estrogen (E2) treatment on SW480 cell survival. Figure S2: The role of estrogen receptor α (ERα) in CRC. Figure S3: E2 role in mitochondrial function of SW480. Figure S4: Identification of potential targets for E2 in CRC. Figure S5: Effect of *RPS2* and *TMEM177* silencing in HCT-116 cells; Table S1: Sequences of primers designed for methylated-specific polymerase chain reaction (MSP). Table S2: Sequences of primers designed for reverse-transcription polymerase chain reaction (RT-PCR). Table S3: Genes with positive and negative correlations with *ESR1* expression in CRC tissues. Table S4: Enrichment among ESR1-correlated genes.
