# Supplementary material for: Estrogen-Induced Hypermethylation Silencing of RPS2 and TMEM177 Inhibits Energy Metabolism and Reduces the Survival of CRC Cells
Source: Cells. 2026 Jan 9;15(2):124. doi: 10.3390/cells15020124 (PMC12839088; doi:10.3390/cells15020124)
Supplement: Supplementary file 1 [file cells-15-00124-s001.zip › Supplementary Figures .pdf]

Supplementary Figures and Figure Legends

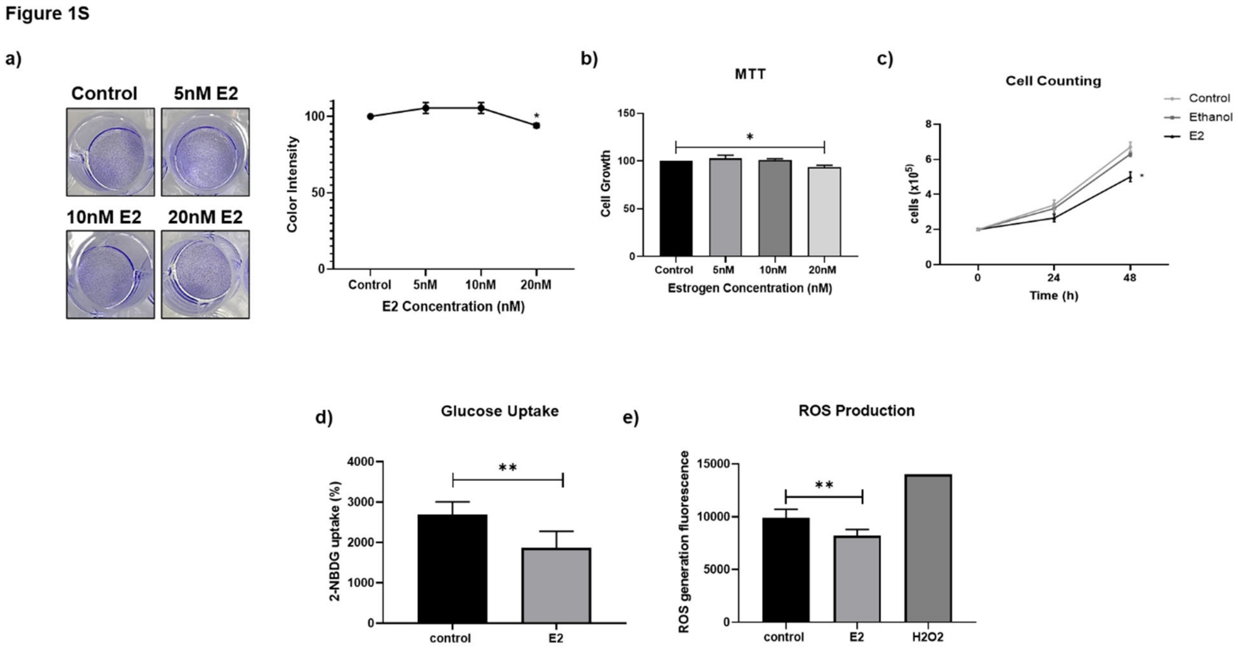

Figure 1S. Effect of estrogen (E2) treatment on SW480 cell survival. (a) SW480 cells were stained with crystal violet (CV) to measure cell proliferation. (b) SW480 cell survival was measured using (3-4,5-dimethyl-2-thiazolyl)-2,5-diphenyl-2-H-tetrazolium bromide (MTT). (c) SW480 cells were stained using trypan blue and counted under the microscope following 20 nM E2 treatment. (d) 2-[N-(7-nitrobenz-2-oxa-1,3-diazol-4-yl) amino]-2-deoxy-glucose (2-NBDG) uptake level was measured using Flow Cytometry in SW480 treated with 20 nM E2. (e) Reactive Oxygen Species (ROS) generation was measured by the 2′,7′-Dichlorofluorescin diacetate (DCFDA) assay.

Figure 2S

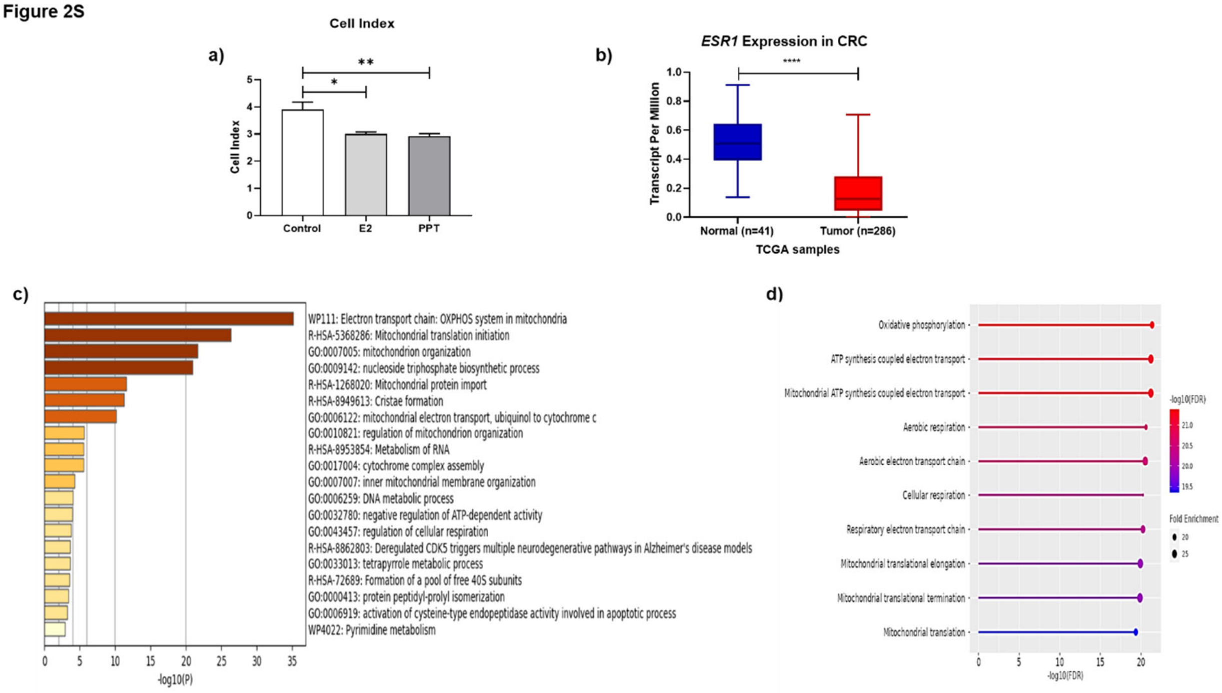

Figure 2S. The role of estrogen receptor α (ERα) in CRC. (a) Real-Time Cell Analysis (RTCA) was used to measure real-time cell proliferation (RTCA) in HCT-116 cells treated with 20 nM E2 and propyl pyrazole triol (PPT), selective ERα agonist. (b) *ESR1* expression in CRC tissue compared to normal tissue. (c) Pathway enrichment analysis of genes negatively correlated with *ESR1* expression in CRC using Metascape database. (d) Pathway enrichment analysis of genes negatively correlated with *ESR1* expression in CRC using ShinyGO v0.741 database.

Figure 3S

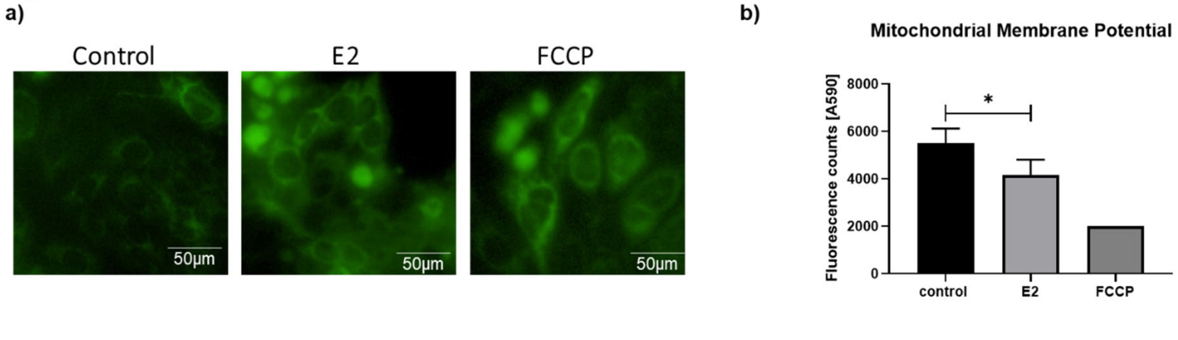

Figure 3S. E2 role in mitochondrial function of SW480. (a) The depolarization of mitochondrial membrane potential (MMP) was observed (the magnification for the figure is 20×) after treating SW480 cells with 20nM E2 . (b) Quantitative analysis of the MMP at 590 nm.

Figure 4S

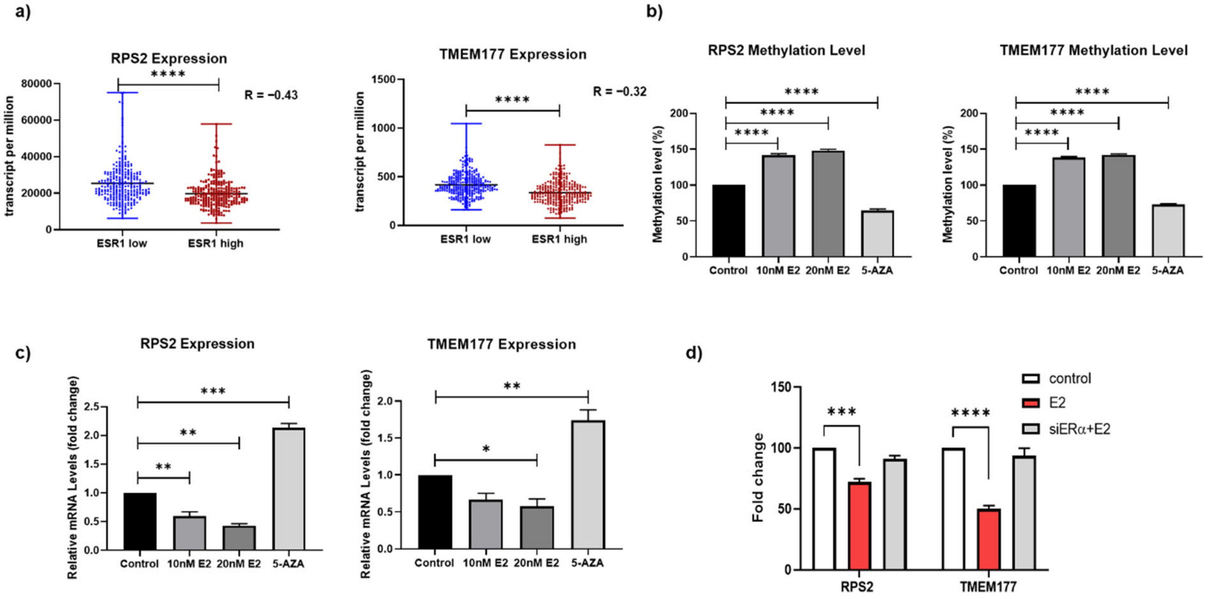

Figure 4S. Identification of potential targets for E2 in CRC. (a) Expression of *RPS2* and *TMEM177* in CRC patients with high *ESR1* expression versus low *ESR1* expression. (b) *RPS2* and *TMEM177* methylation level following 10 and 20 nM E2 and 5-Aza-2-deoxycytidine (Aza) treatments in SW480 cells. (c) *RPS2* and *TMEM177* mRNA expression following 10 and 20 nM E2 and Aza treatments in SW480 cells. (d) Western blot quantifications of the expression of RPS2 and TMEM177 upon E2 treatment with/without ESR1 knockdown in HCT-116 cells.

Figure 5S

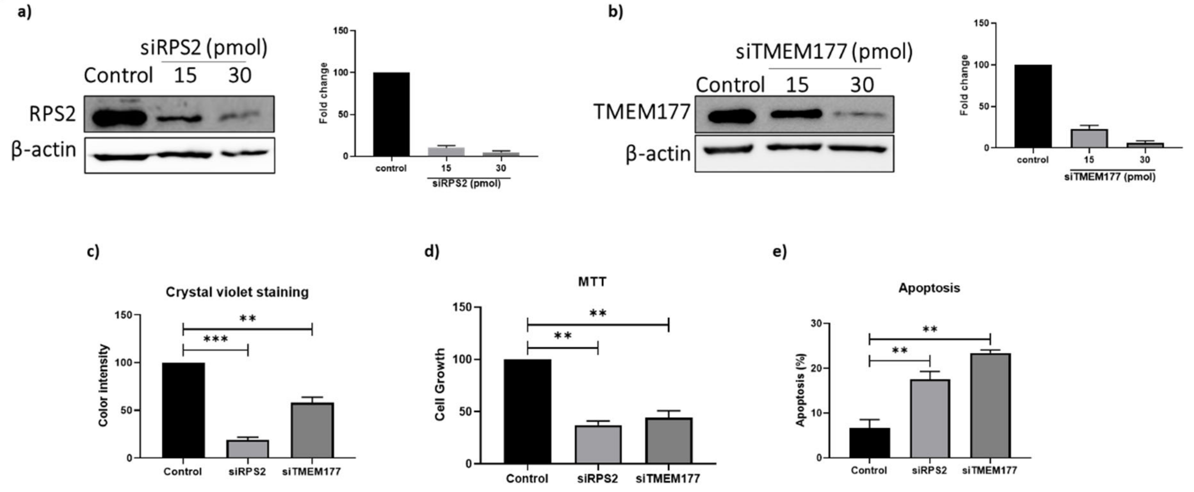

Figure 5S. Effect of *RPS2* and *TMEM177* silencing in HCT-116 cells. (a) Western blot was used to confirm the knockdown of RPS2 using two concentrations of siRPS2 15 and 30 pmol. (b) Western blot was used to confirm the knockdown of TMEM177 using two concentrations of siTMEM177 15 and 30 pmol. (c) Quantification of color intensity of CV staining following the knockdown of RPS2 and TMEM177 in HCT-116 cells. (d) MTT was used to analyze cell viability following RPS2 and TMEM177 knockdown from HCT-116 cells. (e) Quantification of apoptotic cells following RPS2 and TMEM177 knockdown from HCT-116 cells.
