# Supplementary material for: Estrogen-Induced Hypermethylation Silencing of RPS2 and TMEM177 Inhibits Energy Metabolism and Reduces the Survival of CRC Cells
Source: Cells. 2026 Jan 9;15(2):124. doi: 10.3390/cells15020124 (PMC12839088; doi:10.3390/cells15020124)
Supplement: Supplementary file 1 [file cells-15-00124-s001.zip › Supplementary Tables of estrogen paper.pdf]

Table 1S. Sequences of primers designed for methylated-specific polymerase chain reaction (MSP)

MSP	F/R	Sequence (5' > 3')	Tm (ºC)	Product (bp)
RPS2-Methylated	F	ATAGTTATTTTTCGGGTGGATTTTC	66.0	131
	R	CGAACTCTAAATACTCTTACCCGTC	66.0	
RPS2-Unmethylated	F	TAGTTATTTTTTGGGTGGATTTTTG	65.4	130
	R	CAAACTCTAAATACTCTTACCCATC	65.4	
TMEM177-Methylated	F	ACTCTAAACCCCTAAACTACTCCG	69.2	192
	R	GAGTTTCGTTTTTCGAAGTTTTC	69.2	
TMEM177-UnMethylated	F	CTCTAAACCCCTAAACTACTCCACA	66.9	191
	R	GAGTTTTGTTTTTTGAAGTTTTTGT	66.9	

Table 2S. Sequences of primers designed for reverse-transcription polymerase chain reaction (RT-PCR).

Gene	F/R	Sequence (5' > 3')	Tm (ºC)	Product (bp)
RPS2	F	GGCCTCTCTCAAGGATGAGGT	59.8	100
RPS2	R	GTCCCCGATAGCAACAAATGC	57.8	
TMEM177	F	TTCCAATCTCGTACCACCTCT	55.9	120
TMEM177	R	GGAACACCTATGTCCTGTAGCA	58.1	
GAPDH	F	CCAGGTGGTCTCCTCTGACTTC	60.6	108
GAPDH	R	ACATACCAGGAAATGAGCTTGACA	56.7	
